# Molecular Surveillance of *Plasmodium vivax* and *Plasmodium falciparum* Drug Resistance Genes in the Republic of Korea: 2022–2025

**DOI:** 10.3390/pathogens15050508

**Published:** 2026-05-08

**Authors:** Haneul Jung, Hyun-Il Shin, Bora Ku, Esther Park, Myoung-Ro Lee, Jung-Won Ju, Hee-Il Lee

**Affiliations:** 1 Division of Vectors and Parasitic Diseases, Korea Disease Control and Prevention Agency, 187 Osongsaengmyeong 2-ro, Osong-eup, Heungdeok-gu, Cheongju 28159, Chungcheongbuk-do, Republic of Korea; sky123664@korea.kr (H.J.); violet735@korea.kr (B.K.); tmej7@korea.kr (E.P.); blackcg@korea.kr (M.-R.L.); jupapa@korea.kr (J.-W.J.); 2 Division of Zoonotic and Vector Borne Disease Control, Korea Disease Control and Prevention Agency, 187 Osongsaengmyeong 2-ro, Osong-eup, Heungdeok-gu, Cheongju 28159, Chungcheongbuk-do, Republic of Korea; hishin@korea.kr; 3 Division of Laboratory Diagnosis and Analysis, Capital Regional Center for Disease Control and Prevention, Korea Disease Control and Prevention Agency, 131 Yeongjong Haeannam-ro 321beon-gil, Jung-gu, Incheon 22382, Republic of Korea

**Keywords:** malaria, drug resistance, *Pvmdr-1*, *Pfmdr-1*, *PfK13*, *Pfcyt-b*, Republic of Korea

## Abstract

In the Republic of Korea (ROK), chloroquine is the first-line treatment for *Plasmodium vivax* (*P. vivax*), and primaquine is also prescribed to prevent relapse. For *Plasmodium falciparum* (*P. falciparum*), atovaquone-proguanil, pyronaridine-artesunate, and mefloquine are recommended. This study monitored drug resistance genes in blood samples collected from patients with *P. vivax* (soldiers, relapsed, and imported) and *P. falciparum* (soldiers and civilians) between 2022 and 2025. In *P. vivax*, several *Pvmdr-1* mutations showed high prevalence, with S515R, G698S, L845F, M908L, T958M, and F1076L observed in nearly all isolates, while T529 and E1233 mutation showed moderate prevalence. S513R, Y541C, A829V, and S1358 mutation were occasionally identified, all of which were imported or soldiers receiving prophylactic chemotherapy. Significant differences in mutation prevalence were identified among groups (*p* < 0.05). However, no significant temporal trends were observed. In *P. falciparum*, the Y184F mutation in *Pfmdr-1* was highly prevalent, and A675V mutation in *PfK13* was detected only in soldiers. Group comparisons revealed significant differences (*p* < 0.05), whereas temporal trends were not observed. Because drug resistance gene mutations can be closely linked to treatment, surveillance of drug resistance genes is expected to contribute to malaria eradication in the ROK through providing basic information related to treatment.

## 1. Introduction

Malaria is a serious disease that poses a significant threat to public health worldwide. It is transmitted from bites by female *Anopheles* mosquitoes infected with *Plasmodium* spp. [1]. Five species of malaria are known to infect human: *Plasmodium falciparum* (*P. falciparum*), *Plasmodium vivax* (*P. vivax*), *Plasmodium malariae* (*P. malariae*), *Plasmodium ovale* (*P. ovale*), and *Plasmodium knowlesi* (*P. knowlesi*) [2]. *P. falciparum* is primarily prevalent in Africa, with occasional outbreaks of *P. malariae* and *P. ovale*. *P. vivax* is prevalent in Asia and South America, whereas *P. knowlesi* has only been detected in parts of Malaysia and Indonesia [3]. In the Republic of Korea (ROK), malaria is prevalent only in certain areas (Gangwon-do, northern Gyeonggi-do, and Incheon) bordering the Democratic People’s Republic of Korea (DPRK). Approximately 700 cases of *P. vivax* infection occur annually and all other malaria cases are imported [4,5]. Additionally, vivax malaria (malaria caused by infection with *P. vivax*) in the ROK has a long relapse period (up to 2 years) [6].

Chloroquine is used as the first-line treatment for vivax malaria in the ROK, and primaquine is prescribed in combination to prevent relapse. For imported malaria cases, atovaquone-proguanil, pyronaridine-artesunate, and mefloquine are prescribed after confirming epidemiological information, such as the country of origin or travel history of the patient [7]. Chloroquine resistance was first reported in Papua New Guinea [8] and eventually spread worldwide [9,10,11]. The molecular mechanisms underlying chloroquine resistance in vivax malaria are unclear, but it has been reported that high polymorphism of the *P. vivax* multidrug resistance gene (*Pvmdr-1*), particularly the Y976F mutation, is associated [12].

*P. falciparum* multidrug resistance gene (*Pfmdr-1*) mutations, such as N86Y, Y184F, S1034C, N1042D, and D1246Y mutation, are known to modulate chloroquine resistance [13]. However, this depends on the genetic background of the parasite and drug pressure [14]. Artemisinin resistance in *P. falciparum* infection is associated with the kelch13 (K13) propeller domain, and mutations, such as Y493H, R539T, I543T, and C580Y, have been reported to be associated with delayed parasitic clearance [15]. Resistance to atovaquone or atovaquone-proguanil hydrochloride combination therapy arises from Y268S/C/N mutations in the *cytochrome b* gene (*cyt-b*) [16].

Malaria drug resistance is a critical problem that reduces the effectiveness of treatment and increases mortality and patient burden [17]. It is currently a key obstacle in disease control and eradication efforts, with the spread of resistant strains leading to treatment failure, persistent infections, and increased mortality [18]. To this end, malaria drug resistance gene surveillance is a crucial tool for tracking changes in drug susceptibility and provides useful information for national malaria eradication policy [19]. Furthermore, effective preemptive responses through continuous monitoring are cardinal in preventing treatment failure because of drug resistance mutations [20].

Therefore, we conducted a continuous surveillance of antimalarial drug resistance genes of *P. vivax* and *P. falciparum* in the ROK from 2022 to 2025. We also analyzed statistical comparisons across groups and evaluation of temporal trends to provide a more comprehensive understanding of mutation patterns in the ROK. This study aims to provide the foundational evidence necessary to support and accelerate national malaria eradication efforts.

## 2. Materials and Methods

### 2.1. Ethics Statement

This study is exempt from IRB (Institutional Review Board) review because personal identification information of the patient was anonymized, and the results obtained through the study are unrelated to the individual genetic characteristics of the patient. (Bioethics and Safety Act Article 36 and Enforcement Rule of Bioethics and Safety Act Article 33(1)).

### 2.2. Sample Collection and Malaria Diagnosis

Residual specimens of blood samples were collected from patients with malaria who requested a diagnosis from the Division of Vectors and Parasitic Diseases at the KDCA. Malaria was confirmed by microscopic examination of stained blood slides and nested polymerase chain reaction (nested-PCR) [21,22]. Resource samples obtained from malaria case notifications from local public healthcare centers and hospitals (military and civilians) nationwide were also used in this study. A total of 260 *Plasmodium vivax* cases (186 soldiers, 69 relapse cases, and 5 imported cases) and 76 *Plasmodium falciparum* cases (27 soldiers and 49 civilians) were included in the analysis.

Herein, we selected patients with vivax malaria in the ROK who were considered at high risk of drug resistance. The patients were divided into three groups: soldiers receiving prophylactic chemotherapy, patients with relapse vivax malaria, and imported cases of vivax malaria. Soldiers who relapsed were classified separately because these groups were defined as high-risk based on their potential exposure to antimalarial drugs and the associated selective pressure for drug-resistant parasites, including repeated drug exposure (soldiers receiving chemoprophylaxis), possible incomplete parasite clearance (relapse cases), and prior treatment in endemic regions (imported cases). For falciparum malaria (malaria caused by infection with *P. falciparum*), patients were divided into two groups: soldiers with a history of overseas deployment of the armed forces and civilians with a history of overseas travel.

For *P. vivax* relapse, blood samples were collected during both the first and second episodes. Relapse was confirmed by epidemiological data and molecular confirmation of identical subtypes of four genes (apical membrane antigen 1—N1, N2, N3, and N4 subtype, circumsporozoite protein—K1, K2, K3, and K4 subtype, duffy binding protein—T1, T2, T3, and T4 subtype, and merozoite surface protein—recombinant, Sal-a, Sla-b, Sal-c, Sal-R, B-1, B-2, and B-recombinant subtype).

### 2.3. Drug Resistance Gene Amplification

DNA was extracted from 200 µL of blood samples using a QIAamp DNA blood mini kit (Qiagen, Valencia, CA, USA) according to the manufacturer’s protocol (Available online: https://www.qiagen.com/us/resources/kithandbook/hb-0329-007-hb-qiaamp-dna-mini-blood-mini-0725-ww, accessed on 20 April 2026). DNA amplification was performed using AccuPower PCR PreMix (Bioneer, Daejeon, Korea). Primers for amplifying *Pvmdr-1* [12,23,24,25], *Pfmdr-1* [26,27], *PfK13* [28,29,30], and *Pfcyt-b* [31,32] sequences were obtained from previous studies or created in-house. The primers used are listed in Table 1 and the PCR cycling conditions are listed in Table 2.

### 2.4. Sequence Analysis

The size of the amplified PCR products was confirmed using a QiAxcel capillary electrophoresis system (Qiagen, Valencia, CA, USA). Sanger sequencing was performed to analyze the DNA sequences using a commercial sequencing service (SolGent—Daejeon, Republic of Korea). The confirmed DNA sequences were converted to amino acids using Expasy (https://web.expasy.org/translate/, accessed on 21 March 2025) and aligned using the BioEdit 7.2 software (Tom Hall, North Carolina State University, Raleigh, NC, USA) to identify mutations.

### 2.5. Statistical Analysis

Statistical analyses were performed to evaluate differences in mutation prevalence across groups and over time. Mutation frequencies were expressed as proportions, and comparisons between groups (soldier, relapse, and imported cases) were conducted using the chi-square test or Fisher’s exact test when expected counts were less than five. To assess temporal trends, logistic regression analysis was performed with year treated as an ordinal variable to evaluate changes in mutation prevalence from 2022 to 2025. All statistical tests were two-tailed, and a *p*-value of <0.05 was considered statistically significant. Statistical analyses were performed using R software version 4.4.1.

## 3. Results

### 3.1. Polymorphism of the Pvmdr-1 Gene

**Figure 1:**
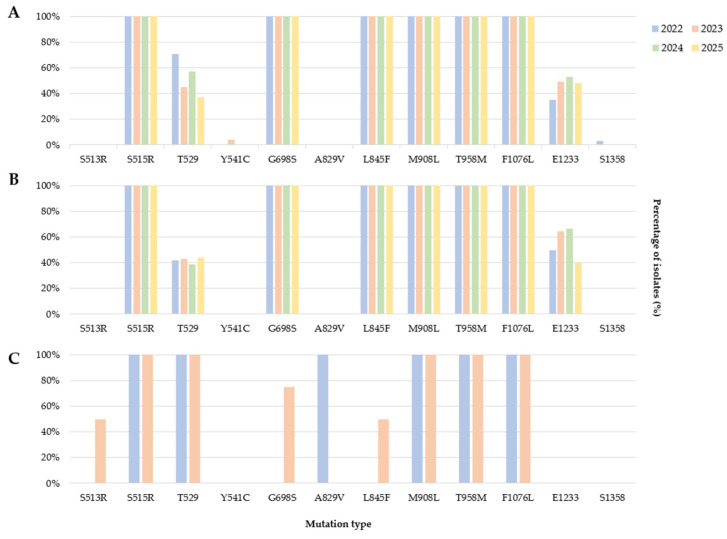
*Plasmodium vivax* multidrug resistance gene (*Pvmdr-1*) mutation ratio in the ROK (2022–2025). (**A**) Soldiers, (**B**) patients who relapsed, and (**C**) imported cases. Mutations that were not detected are excluded from the figure. The bars represent the percentage of isolates carrying each mutation in the indicated group/year.

A total of 260 vivax malaria samples were analyzed (47 in 2022, 67 in 2023, 94 in 2024, and 52 in 2025) (Table 3) and divided into three groups: soldiers, relapsed patients, and imported cases. In the military personnel and relapsed patient groups, six mutations (S515R, G698S, L845F, M908L, T958M, and F1076L) were identified in 100% of cases each year, while T529 and E1233 mutations were detected in approximately 50% of cases (Figure 1). This confirms that sextuple mutations are predominantly found in high-risk patients in the ROK, such as soldiers receiving preventive chemotherapy or patients who have relapsed for whom treatment is not expected to be effective. T529 and E1233 mutation were detected in approximately half of patients harboring octuple mutations. Interestingly, as Y541C and S1358 mutations were rarely detected only in military patients, they appear to be related to prophylactic chemotherapy [24,25]. Regarding imported cases, five mutations, S515R, T529, M908L, T958M, and F1076L, were confirmed in 100% of the cases, and G698S and L845F mutations were confirmed in approximately 50% of the cases. S513R and A829V mutations were ascertained only in imported cases and therefore appear to be mutations that have not yet been established in the ROK. Moreover, other mutations have not yet been confirmed.

### 3.2. Polymorphism of Pfmdr-1, PfK13, and Pfcyt-b Genes

All cases of falciparum malaria identified in the ROK were imported, and drug resistance was influenced by the country of origin of the patient’s infection. This study analyzed 76 falciparum malaria samples (5 in 2022, 30 in 2023, 19 in 2024, and 22 in 2025) (Table 4).

**Figure 2:**
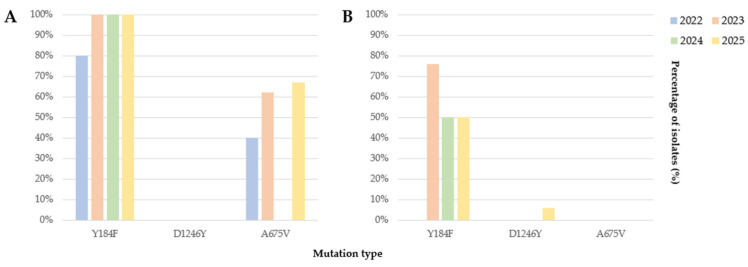
*Plasmodium falciparum* drug resistance gene (*Pfmdr-1*, *PfK13*, and *Pfcyt-b*) mutation ratio in the ROK (2022–2025). (**A**) Soldiers and (**B**) civilians. Mutations that were not detected are excluded from the figure. The bars represent the percentage of isolates carrying each mutation in the indicated group/year.

Mutations targeted for surveillance included 7 in *Pfmdr-1*, 14 in *PfK13*, and 3 in *Pfcyt-b* (Table 1). However, only Y184F and D1246Y mutations in *Pfmdr-1* and A675V mutation in *PfK13* were identified. According to patient epidemiological data, all soldiers infected with falciparum malaria had been deployed to South Sudan, and civilian patients had a history of visiting Africa. The Y184F mutation was identified in both the soldier and civilian groups, with a higher rate in soldiers. The soldier group showed the Y184F mutation in 100% of cases each year, except in 2022, while the civilian group showed the mutation in 50–76% of cases each year (Figure 2). This confirms that the Y184F mutation is spread throughout Africa, including South Sudan. The D1246Y mutation was first revealed in 2025 in travelers who visited Rwanda, highlighting the importance of domestic surveillance of imported falciparum malaria drug resistance genes. The Y184F mutation was identified in both soldiers and civilians, whereas the A675V mutation was found only in soldiers who had been deployed to South Sudan. The A675V mutation was only detected in soldiers, with an annual prevalence range of 0–67%. Continued surveillance is therefore crucial because of high annual variations in prevalence.

### 3.3. Statistical Analysis of the Pvmdr-1, Pfmdr-1, PfK13, and Pfcyt-b Genes

A total of 12 mutations of *Pvmdr-1* were included in the comparative analysis after excluding mutations with no observed occurrences (Table 5). Significant differences in mutation prevalence were observed for six mutations: S513R, T529, G698S, A829V, L845F, and E1233 (*p* < 0.05). No significant differences were identified for the remaining mutations. Temporal trends in *Pvmdr-1* mutation prevalence were evaluated using logistic regression. No statistically significant changes in mutation prevalence were observed over the study period. Although the E1233 mutation showed a marginal increasing trend, this did not reach statistical significance (*p* = 0.08).

In case of *P. falciparum*, significant differences were observed for the Y184F and A675V mutations (*p* < 0.05) in group comparisons (Table 6). The Y184F mutation was more prevalent in soldiers than civilians, whereas the A675V mutation was detected exclusively in soldiers. No significant difference was observed for the D1246Y mutation. Temporal trend analysis using logistic regression showed no statistically significant changes in mutation prevalence over the study period. Although year-to-year fluctuations were observed for Y184F and A675V, these did not demonstrate consistent temporal trends. The D1246Y mutation remained rare throughout the study period.

## 4. Discussion

In the ROK, malaria was eradicated in the 1970s but resurged in the region near the Demilitarized Zone (DMZ) bordering the DPRK in 1993 [33]. Approximately 500–600 cases have been reported in recent years, posing a significant public health problem. Malaria eradication in the ROK remains challenging for several reasons. First, the DPRK is a permanent reservoir [34]. Although human movement across borders is restricted by the DMZ, mosquitoes can freely move back and forth. Genetic analysis has suggested that *P. vivax* in the ROK and DPRK is essentially similar [35]. This indicates that the Korean malarial strain is a transboundary strain. According to World Health Organization (WHO) standards, the continuous cross-border influx of malaria makes it difficult for a single country to achieve eradication [36]. Although border malaria is an issue that requires a coordinated effort between two or more countries, cooperation between the ROK and the DPRK is difficult for political reasons, posing a momentous challenge for malaria eradication in the former. Second, malaria in the ROK has an extremely long latent period [6]. Many strains harbor hypnozoites that can induce long incubation periods of 8–12 months or more, making epidemiological tracking difficult [33]. Patients with hypnozoites are more likely to relapse and are predicted to contribute largely to vivax malaria transmission [37].

Treatment of vivax malaria in the ROK involves a combination of chloroquine (25 mg base/kg), which eliminates blood-stage parasites, and primaquine (3.5 mg base/kg), which eliminates hypnozoites [38]. Although it has been used for more than 30 years, chloroquine is still effective in the ROK [39]. However, the long-term use of the drug and prophylactic chemotherapy administered to soldiers increases the risk of drug resistance. In Southeast Asia, resistance commonly occurs within 10–15 years of chloroquine use. However, the actual rate of resistance varies depending on factors, such as the intensity of drug use, transmission intensity, and parasite population size. Therefore, continuous surveillance is necessary [40]. Research on malarial drug resistance in the DPRK and northeastern China is very limited; however, chloroquine-resistant strains are highly likely to exist in these regions [41]. Malaria in the ROK is closely linked to the malaria epidemic in the DPRK. Therefore, detecting chloroquine-resistant parasite strains at the genetic level is warranted before they cross borders and become entrenched. Furthermore, the WHO suggests that genetic surveillance of drug resistance is crucial in advancing the elimination stage of malaria [36]. Therefore, ongoing genetic surveillance of *Pvmdr-1* is needed in the ROK. As a government agency controlling malaria diagnosis, the KDCA collects specimens representing approximately 80% of the annual malaria cases. Surveillance of antimalarial drug resistance genes is conducted for soldier, relapse, and imported cases, as part of efforts toward malaria elimination.

From 2022 to 2025, analysis of *Pvmdr-1* in patients with vivax malaria classified as high-risk groups subject to drug resistance surveillance, including soldiers, relapsed patients, and imported patients, revealed the presence of six mutations (S515R, G698S, L845F, M908L, T958M, and F1076L) in 97–100% of cases (Figure 1). Furthermore, two mutations (T529 and E1233) were identified in approximately 50% of the cases, confirming that the mutations were endemic in the ROK. The absence of the Y976F mutation in pvmdr-1, a marker associated with chloroquine resistance, may indicate a low prevalence of this specific resistance-associated mutation in the study population. The Y976F mutation, which is highly associated with reduced chloroquine susceptibility, was not found; however, the F1076L mutation, a secondary requirement for Y976F mutation acquisition [42], was detected in 100% of the annual cases. From a two-step mutational trajectory perspective, the Y976F mutation is rarely observed alone and tends to be observed together with the F1076L mutation or appear after the F1076L mutation [43]. This suggests that the Y976F mutation could emerge in the ROK, underscoring the importance of ongoing genetic surveillance. To track the Y976F mutation, a more robust plan will be implemented for the aforementioned high-risk patients, including drug resistance monitoring on a quarterly basis.

The S513R and A829V mutations were revealed only in patients with imported vivax malaria (Table 3). The S513R mutation was also detected in patients from India and Pakistan, a finding epidemiologically consistent with data showing a high incidence in South Asia [44]. The A829V mutation was identified in a patient from Pakistan. This rare mutation is likely a lineage-associated polymorphism rather than a direct cause of resistance [45]. In contrast, the Y541C and S1358 mutations were identified only in soldiers who served in the DMZ (Table 3), which is consistent with previous research [24,25]. Our findings suggest that these mutations reflect a chemoprophylaxis-driven selective sweep in soldiers in the ROK.

All *P. falciparum* isolates ascertained in the ROK were imported. Most patients were epidemiologically confirmed to have a history of travel in Africa. Atovaquone-proguanil, pyronaridine-artesunate, and mefloquine are recommended as primary treatments, while artesunate is used intravenously for the treatment of severe falciparum malaria [38]. Therefore, various *P. falciparum* drug resistance genes, such as *Pfmdr-1* (chloroquine and mefloquine), *PfK13* (pyronaridine-artesunate), and *Pfcyt-b* (atovaquone-proguanil), were investigated from 2022 to 2025 in all patients who had traveled, with only the Y184F and D1246Y mutations of *Pfmdr-1* and the A675V mutation of *PfK13* identified (Table 4).

The most common mutation found in imported cases of *P. falciparum* malaria was the Y184F mutation in *Pfmdr-1*, with a high prevalence of 58–87% annually (Figure 2). This mutation is frequently reported worldwide, and the results are consistent with previous studies [31,46,47,48]. Although it may not be a strong resistance marker on its own, it should be closely monitored, as it can interact with other mutations and exert an impact. Notably, the D1246Y mutation was first detected in a patient with a history of travel to Rwanda in 2025. Although the D1246Y mutation occurs with high frequency in South America, it has rarely been identified in Africa [49,50]. It has also been reported in Rwanda, where it persists at a low rate without being completely eliminated [51]. Although the D1246Y mutation does not confer strong resistance, it warrants attention because it reflects drug selection pressure in artemisinin-based combination therapies, which can influence treatment failure.

The A675V mutation in *PfK13* is recognized by the WHO as being associated with delays in artemisinin treatment. This mutation has been reported at low frequencies in several African countries, including Uganda, Rwanda, Nigeria, and Kenya [52,53]. Although not yet widely established as a dominant resistance mutation, its increasing detection in certain regions suggests a potential emerging marker of artemisinin resistance, highlighting the need for continued molecular surveillance. A notable finding regarding the A675V mutations ascertained in the ROK-imported cases was that all patients were soldiers who had been deployed to South Sudan. This mutation is reported at low levels in Africa and has not been revealed in South Sudan to date [54,55], highlighting the need to monitor deployed soldiers for treatment failure or delays.

This study evaluated mutation patterns associated with antimalarial drug resistance in both *P. vivax* and *P. falciparum*, focusing on differences across population groups and temporal trends over a four-year period. In both species, differences in mutation prevalence were observed across groups, suggesting potential variation in parasite populations or exposure history. However, no significant temporal trends were identified for either species over the study period, indicating that the overall mutation profiles remained relatively stable from 2022 to 2025. Although minor year-to-year fluctuations were observed, these were not consistent and are likely attributable to random variation or differences in sample composition. This study has several limitations. First, the number of imported *P. vivax* cases was small, which may have influenced the statistical significance of group comparisons and potentially exaggerated observed differences. Second, the overall sample size for *P. falciparum*, particularly in certain years, was limited, reducing the statistical power to detect subtle temporal trends. In conclusion, this study demonstrates that while group-specific differences in mutation prevalence exist in both *P. vivax* and *P. falciparum*, the overall mutation patterns remained stable over time. The results of this study highlight the importance of continued molecular surveillance to monitor potential shifts in antimalarial resistance and to better understand the epidemiological dynamics of malaria infections. These findings also have broader implications beyond the ROK. Continuous monitoring of antimalarial drug resistance markers provides essential information for detecting changes in parasite populations and supporting timely public health responses. Such surveillance efforts contribute to strengthening malaria control strategies and may support global efforts toward malaria elimination.

The overall proportion of drug resistance genes for vivax and falciparum malaria in the ROK has not changed significantly over the investigated period; however, a small number of mutations were identified intermittently. However, it should be noted that the presence of molecular markers does not necessarily indicate phenotypic resistance or treatment failure, and further studies are required to confirm their clinical significance. This study was conducted in high-risk groups rather than in all patients during the investigation period. Therefore, it should be noted that this study does not represent the entire population of malaria patients in the ROK. Although chloroquine is still known to be susceptible to vivax malaria in the ROK, continued monitoring is necessary due to climate change, an influx of overseas workers, and increased travel. Monitoring drug resistance is important for malaria control because the emergence of drug resistance can lead to increased treatment delays or failure, potentially creating a new source of infection. Furthermore, the association between the mutations (S513R and A829V) detected only in soldiers and chemoprophylaxis needs to be investigated. As *P. falciparum* malaria occurs exclusively through overseas travel, drug resistance mutations are also influenced by the countries visited. Although no cases of treatment failure exist to date, careful attention is needed because of its high mortality rate. Additionally, for soldiers deployed in South Sudan, identifying cases of delayed onset or treatment delays owing to chemoprophylaxis is warranted. Collaboration with the military is essential for developing treatment strategies that consider drug-resistant mutations of *P. falciparum* malaria and for selecting more effective preventive drugs.

In summary, this study provides updated data on antimalarial drug resistance mutations in the ROK, including statistical comparisons across groups and temporal trend analyses. These findings support ongoing surveillance efforts for malaria control and elimination. The KDCA plans to continue monitoring drug resistance to generate basic data for malaria eradication. Furthermore, follow-up studies are needed to comprehensively analyze various factors known to influence drug activity and metabolism, such as *P. vivax* chloroquine resistance transporter-o [56] and cytochrome P450, family 2, subfamily D, polypeptide 6 [57,58].

## 5. Conclusions

This study provides an updated overview of antimalarial drug resistance markers in *P. vivax* and *P. falciparum* in the ROK from 2022 to 2025. Several mutations were consistently observed, while others were detected only sporadically or not at all, indicating distinct patterns of mutations prevalence within the study population. These finding highlight the importance of continuous molecular surveillance to monitor changes in parasite populations and to support evidence-based malaria control strategies. Such efforts will be essential for guiding effective treatment policies and advancing malaria elimination in the ROK.

## Figures and Tables

**Table 1:** Primers used in the present study.

Gene	Primer		Sequence (5′ to 3′)	PCR-Fragment Size	Mutation
Pvmdr-1	mdr-2F		CAGCCTGAAGATTTAGAAGCCTT	539	S513R, S515R, T529, Y541C, I636T
	mdr-2R		CATCCACGTCCACAGTGGAAC		
	1981F		GTGGACGTGGATGTGCTGGGCGA	900	G698S, A829V, L845F, M908L
	2880R		CATGACCGTACTCACAAGGAAGA		
	mdr-3F		GGATAGTCATGCCCCAGGATTG	604	T958M, Y976F, K997R, L1022, F1076L
	mdr-3R		CATCAACTTCCCGGCGTAGC		
	mdr-7F		GATGAGCCTGCTGATGCGATTCTAC	745	E1233, S1358, K1393N, E1396
	mdr-5R		ATATACGCCGTCCTGCACCGAG		
Pfmdr-1	1st PCR	Pfmdr-86-1F	TTAAATGTTTACCTGCACAACATAGAAAATT	612	N86Y, E130K, Y184F
		Pfmdr-86-1R	CTCCACAATAACTTGCAACAGTTCTTA		
	2nd PCR	Pfmdr-86-2F	TGTATGTGCTGTATTATCAGGA	526	
		Pfmdr-86-2R	CTCTTCTATAATGGACATGGTA		
	1st PCR	Pfmdr-1034-1F	AATTTGATAGAAAAAGCTATTGATTATAA	880	S1034C, N1024D, V1109I, D1246Y
		Pfmdr-1034-1R	TATTTGGTAATGATTCGATAAATTCATC		
	2nd PCR	Pfmdr-1034-2F	GAATTATTGTAAATGCAGCTTTA	799	
		Pfmdr-1034-2R	GCAGCAAACTTACTAACACG		
PfK13	1st PCR	K13-0F	GGGAATCTGGTGGTAACAGC	2097	R471, Y493H, V494I, S522C, V534A, R539T, I543T, R575, A578S, C580Y, F583L, V589I, R622I, A675V
		K13-0R	CGGAGTGACCAAATCTGGGA		
	2nd PCR	K13-1F	GCCTTGTTGAAAGAAGCAGA	849	
		K13-1R	GCCAAGCTGCCATTCATTTG		
Pfcyt-b	cyt-b1		CTCTATTAATTTAGTTAAAGCACA	939	Y268S/C/N
	cyt-b2		ACAGAATAATCTCTAGCACC		

The *Pfmdr-1* and *PfK13* genes are amplified using nested-PCR.

**Table 2:** PCR cycling conditions.

Step	*Pvmdr-1*			*Pfmdr-1* and *PfK13*			*Pfcyt-b*		
Pre-denaturation	95 °C	5 min	1 cycle	95 °C	5 min	1 cycle	95 °C	5 min	1 cycle
Cycle	95 °C	1 min	35 cycles	95 °C	30 s	35 cycles	95 °C	50 s	50 cycles
	58 °C	30 s		58 °C	30 s		55 °C	50 s	
	72 °C	1 min		72 °C	1 min		72 °C	1 min	
Final extension	72 °C	10 min	1 cycle	72 °C	10 min	1 cycle	72 °C	10 min	1 cycle

*Pvmdr-1* and *Pfcyt-b* were amplified using conventional PCR. *Pfmdr-1* and *PfK13* were amplified using nested-PCR. With nested-PCR, 1st- and 2nd-stage conditions were the same.

**Table 3:** *Plasmodium vivax* multidrug resistance mutations in the ROK (2022–2025).

Year	Group	*N*	S513R	S515R	T529	Y541C	G698S	A829V	L845F	M908L	T958M	F1076L	E1233	S1358
2022	Soldier	34	-	34	24	-	34	-	34	34	34	34	12	1
	Relapse (Soldier)	12 (1)	-	12 (1)	5 (1)	-	12 (1)	-	12 (1)	12 (1)	12 (1)	12 (1)	6 (1)	-
	Imported	1	-	1	1	-	-	1	-	1	1	1	-	-
2023	Soldier	49	-	49	22	2	49	-	49	49	49	49	24	-
	Relapse	14	-	14	6	-	14	-	14	14	14	14	9	-
	Imported	4	2	4	4	-	3	-	2	4	4	4	-	-
2024	Soldier	76	-	76	43	-	76	-	76	76	76	76	40	-
	Relapse	18	-	18	7	-	18	-	18	18	18	18	12	-
2025	Soldier	27	-	27	10	-	27	-	27	27	27	27	13	-
	Relapse (Soldier)	25 (1)	-	25 (1)	11 (0)	-	25 (1)	-	25 (1)	25 (1)	25 (1)	25 (1)	10 (1)	-
Total		260	2	260	133	2	258	1	257	260	260	260	126	1

The study group includes soldiers receiving chemoprophylaxis, relapse, and imported cases. The total number of samples (*N*) and mutations in each group is indicated in the table. Because I636T, Y976F, K997R, L1022, K1393N, and E1396 mutations were not confirmed, they are excluded from the table.

**Table 4:** *Plasmodium falciparum* drug resistance mutations in the ROK (2022–2025).

Gene				*Pfmdr-1*		*PfK13*
Year	Group	*N*	Traveled Country	Y184F Mutation	D1246Y Mutation	A675V Mutation
2022	Soldier	5	South Sudan	4	-	2
2023	Soldier	13	South Sudan	13	-	8
	Civilian	17	**Gabon, Ghana**, **Guinea**, Liberia, **Nigeria**, **Republic of South Africa**, **Sudan**, **Tanzania**, **Uganda**	13	-	-
2024	Soldier	3	South Sudan	3	-	-
	Civilian	16	**Burkina Faso**, **Cameroon**, **Cote d’Ivoire**, DR Congo, **Ethiopia**, **Ghana**, **Japan**, Kenya, Madagascar, Nigeria, Republic of South Africa, **Tanzania**, Uganda	8	-	-
2025	Soldier	6	South Sudan	6	-	4
	Civilian	16	Angola, Burkina Faso, Cameroon, **Cote d’Ivoire**, Equatorial Guinea, **Nigeria**, Rwanda, Senegal, **Uganda**	8	1	-
Total		76		55	1	14

Mutations that were not detected are excluded from the table. All soldiers had been deployed to South Sudan, and civilians had traveled to Africa. Countries in which we detected the Y184F mutation are indicated in bold.

**Table 5:** Comparison of *Pvmdr-1* mutation prevalence among groups (2022–2025 pooled).

Gene	Mutation	Soldier (n/N, %)	Relapse (n/N, %)	Imported (n/N, %)	*p*-Value
*Pvmdr-1*	S513R	0/186 (0.0)	0/69 (0.0)	2/5 (40.0)	0.0007
	S515R	186/186 (100)	69/69 (100)	5/5 (100)	>0.99
	T529	99/186 (53.2)	29/69 (42.0)	5/5 (100)	0.018
	Y541C	2/186 (1.1)	0/69 (0.0)	0/5 (0.0)	>0.99
	G698S	186/186 (100)	69/69 (100)	3/5 (60.0)	0.0003
	A829V	0/186 (0.0)	0/69 (0.0)	1/5 (20.0)	0.019
	L845F	186/186 (100)	69/69 (100)	2/5 (40.0)	<0.0001
	M908L	186/186 (100)	69/69 (100)	5/5 (100)	>0.99
	T958M	186/186 (100)	69/69 (100)	5/5 (100)	>0.99
	F1076L	186/186 (100)	69/69 (100)	5/5 (100)	>0.99
	E1233	89/186 (47.8)	37/69 (53.6)	0/5 (0.0)	0.032
	S1358	1/186 (0.5)	0/69 (0.0)	0/5 (0.0)	>0.99

Values are presented as number of mutation-positive samples over total samples (percentage). *p*-values were calculated using the chi-square test or Fisher’s exact test, as appropriate.

**Table 6:** Comparison of *Pfmdr-1* and *PfK13* mutation prevalence among groups (2022–2025 pooled).

Gene	Mutation	Soldier (n/N, %)	Civilian (n/N, %)	*p*-Value
*Pfmdr-1*	Y184F	26/27 (96.3)	29/49 (59.2)	0.0008
	D1246Y	0/27 (0.0)	1/49 (2.0)	>0.99
*PfK13*	A675V	14/27 (51.9)	0/49 (0.0)	<0.0001

Values are presented as number of mutation-positive samples over total samples (percentage). *p*-values were calculated using the chi-square test or Fisher’s exact test, as appropriate.

## Data Availability

The original contributions presented in this study are included in the article. Further inquiries can be directed to the corresponding author.

## Abbreviations

The following abbreviations are used in this manuscript:

DMZ	Demilitarized Zone
DPRK	Democratic People’s Republic of Korea
KDCA	Korea Disease Control and Prevention Agency
PCR	Polymerase chain reaction
ROK	Republic of Korea
WHO	World Health Organization
